# Vaccination with Recombinant RNA Replicon Particles Protects Chickens from H5N1 Highly Pathogenic Avian Influenza Virus

**DOI:** 10.1371/journal.pone.0066059

**Published:** 2013-06-10

**Authors:** Stefan J. Halbherr, Terza Brostoff, Merve Tippenhauer, Samira Locher, Marianne Berger Rentsch, Gert Zimmer

**Affiliations:** 1 Institute of Virology and Immunology (IVI), Mittelhäusern, Switzerland; 2 Department of Pathology, Microbiology and Immunology, School of Veterinary Medicine, University of California Davis, Davis, California, United States of America; Thomas Jefferson University, United States of America

## Abstract

Highly pathogenic avian influenza viruses (HPAIV) of subtype H5N1 not only cause a devastating disease in domestic chickens and turkeys but also pose a continuous threat to public health. In some countries, H5N1 viruses continue to circulate and evolve into new clades and subclades. The rapid evolution of these viruses represents a problem for virus diagnosis and control. In this work, recombinant vesicular stomatitis virus (VSV) vectors expressing HA of subtype H5 were generated. To comply with biosafety issues the G gene was deleted from the VSV genome. The resulting vaccine vector VSV*ΔG(HA) was propagated on helper cells providing the VSV G protein *in trans*. Vaccination of chickens with a single intramuscular dose of 2×10^8^ infectious replicon particles without adjuvant conferred complete protection from lethal H5N1 infection. Subsequent application of the same vaccine strongly boosted the humoral immune response and completely prevented shedding of challenge virus and transmission to sentinel birds. The vaccine allowed serological differentiation of infected from vaccinated animals (DIVA) by employing a commercially available ELISA. Immunized chickens produced antibodies with neutralizing activity against multiple H5 viruses representing clades 1, 2.2, 2.5, and low-pathogenic avian influenza viruses (classical clade). Studies using chimeric H1/H5 hemagglutinins showed that the neutralizing activity was predominantly directed against the globular head domain. In summary, these results suggest that VSV replicon particles are safe and potent DIVA vaccines that may help to control avian influenza viruses in domestic poultry.

## Introduction

Outbreaks of highly pathogenic influenza viruses (HPAIV) in chickens and turkeys have led to profound economical losses in many regions, including Italy, The Netherlands, Egypt, Mexico, and Southeast Asia [1], [2]. A common feature of HPAIV is the presence of a multi-basic cleavage site that is recognized by furin-like proteases [3]. As a consequence of the ubiquitous expression of these proteases, HPAIVs cause systemic multi-organ disease in poultry. This disease is historically known as “fowl plague” reflecting its rapid spread and high mortality rates of up to 100%. Although several AIV subtypes could potentially mutate to become highly pathogenic [4], only HPAIV of subtypes H5 and H7 have been detected in nature so far. It is generally believed that LPAIVs occasionally evolve into HPAIVs when circulating in domestic poultry [1], [5].

Direct transmission of AIVs from birds to humans is rare. A well-known barrier to transmission is the lack of appropriate receptors in the human upper respiratory tract [6]. Nevertheless, human infections with H7 and H5 AIV have occurred in the past, usually following direct exposure of persons to infected poultry and high virus doses [7], [8]. HPAIV of subtype H5N1 can cause fatal disease in humans, but fortunately human-to-human transmission of H5N1 has not been observed yet. Nevertheless, further adaptation of H5N1 to humans or reassortment with human influenza viruses may result in aerosol-transmittable viruses with pandemic potential [9]–[12].

Vaccination of domestic poultry may help to control HPAIV and to reduce both economical losses in poultry farming and potential zoonotic transmission to humans. Inactivated influenza virus vaccines have been used in the past to control H5N1 epizootics in poultry [13]. These vaccines have the advantage of being safe, but they also come with a number of shortcomings. For example, inactivated vaccines have to be repeatedly applied to induce full-protective and long-lasting immune responses in poultry [14]. Because inactivated influenza virus vaccines do not provide danger signals that would sufficiently trigger innate immunity, they are often formulated and applied with adjuvants [15]–[17]. Inactivated influenza virus vaccines are usually administered via the intramuscular route making mass vaccination of poultry labor-intensive and expensive. A general problem for vaccination is the antigenic drift of influenza viruses. Full protection may be achieved only if the selected vaccine strain closely matches the major antigens of currently circulating field viruses. However, if vaccination provides only partial protection, immunized animals may appear healthy but may shed virus, which could lead to unnoticed virus spread.

If vaccines are applied to control AIV outbreaks in poultry, they should allow serological differentiation of infected from vaccinated animals (DIVA). Inactivated DIVA vaccines containing a different NA subtype have been used to control AIV outbreaks in Italy [18], [19]. However, such vaccines would not be accepted for the general prophylactic vaccination of poultry against H5 and H7 viruses because of potential restrictions in trading of poultry and poultry products. Live-attenuated influenza virus vaccines can induce broader and longer lasting immune responses than inactivated vaccines [20]. However, the possible formation of reassortants between vaccine and field viruses argues against the use of live-attenuated AIV vaccines.

Recent studies have shown that a propagation-incompetent vesicular stomatitis virus (VSV) vector encoding HA_H7_ protects chickens from challenge infection with HPAIV of subtype H7N1 [21]. In the present work, the capacity of recombinant VSV replicon particles to induce protective immunity against HPAIV of subtype H5N1 was evaluated in chickens. Specifically, the vaccine dose and the number of applications required to induce protection and to reduce virus shedding was studied. Furthermore, sera from immunized chickens were analyzed for virus neutralizing activity against a limited number of H5 viruses from different clades. Finally, the compatibility of the vaccine with the DIVA principle was examined.

## Materials and Methods

### Ethics statement

Animal trials were were performed in compliance with the Swiss animal protection law and approved by the animal welfare committee of the Canton of Berne (authorization number 76/10).

### Cells

BHK-21 cells were obtained from the German Cell Culture Collection (DSZM, Braunschweig; Germany) and grown in Earle's minimal essential medium (MEM; Life Technologies, Carlsbad, CA) supplemented with 5% fetal bovine serum (FBS; Biowest). BHK-G43, a transgenic BHK-21 cell clone expressing the VSV G protein in a regulated manner, was maintained as described previously [22]. Madin-Darby canine kidney (MDCK) cells (type I) were provided by Georg Herrler (TiHo Hannover) and cultured with MEM and 5% FBS. Vero cells (C1008) were purchased from the American Type Culture Collection (Manassas, VA) and maintained in Dulbecco's modified Eagle medium (DMEM; Life Technologies; Carlsbad CA) supplemented with 5% FBS.

### Viruses

HPAIV A/chicken/Yamaguchi/7/2004 (H5N1) [23], HPAIV A/whooper swan/Mongolia/3/2005 (H5N1), and LPAIV A/duck/Hokkaido/Vac-1/2004 (H5N1) [24] were kindly provided by Yoshihiro Sakoda, Hokkaido University, Sapporo, Japan. The following LPAIV were kindly provided by Timm Harder (Friedrich-Löffler-Institut, Riems-Greifswald, Germany): A/duck/Potsdam/1402/86 (H5N2), A/duck/Potsdam/2216/84 (H5N6), A/duck/Potsdam/619/85 (H5N2), A/ostrich/Germany/R5-10/03 (H5N2), A/mallard/Föhr/Wv1310-13/03 (H5N2), and A/teal/Föhr/Wv1378-79/03 (H5N2). HPAIV A/Cygnus olor/Italy/742/2006 (H5N1) and LPAIV A/duck/Italy/1447/2005 (H1N1) were kindly provided by William Dundon (IZSV Istituto Zooprofilattico Sperimentale delle Venezie, Venice, Italy). NIBRG-14, a 2∶6 reassortant virus between A/Vietnam/1194/2004 (H5N1) and A/Puerto Rico/8/1934 (H1N1) was provided by J. Robertson (National Institute for Biological Standards and Control, UK). The HA of this virus contains a modified cleavage site [25]. All viruses were propagated in the allantoic cavity of 10-day old embryonated specific pathogen-free (SPF) chicken eggs for 2 days at 37°C. HA cDNA was determined and deposited at the EMBL nucleotide sequence database for the following viruses: HF563054 for A/duck/Italy/1447/2005 (H1N1), HF563055 for A/duck/Potsdam/619/1985 (H5N2), HF563056 for A/mallard/Foehr/Wv1310-13/2003 (H5N2), HF563057 for A/ostrich/Germany/R5-10/2006 (H5N3), and HF563058 for A/teal/Foehr/Wv1378-79/2003 (H5N2).

Influenza viruses were titrated on MDCK cells either in the absence (for titration of HPAIV) or presence (for titration of LPAIV) of 1 µg/ml of acetylated trypsin (Sigma-Aldrich, St. Louis, MO). At 72 hours post infection (p.i.), the cells were washed with PBS and fixed with 10% formalin containing 0.1% (w/v) crystal violet. The plates were washed with tap water to remove excess crystal violet and dried. If LPAIV did not induce obvious cytopathic effects, infected cells were visualized by immunostaining with an anti-NP monoclonal antibody (clone H16-L10-4R5; ATCC, HB-65) according to a previously published protocol [26]. Virus titers were calculated according to the Spearman-Kärber method [27], [28] and expressed as 50% tissue culture infectious doses (TCID_50_/ml).

### Construction of plasmids

The cDNAs encoding HA_H5_ of HPAIV A/chicken/Yamaguchi/7/2004 (clade 2.5) and LPAIV A/duck/Hokkaido/Vac-1/2004 (classical clade) were kindly provided by Y. Sakoda, Sapporo, Japan (EMBL/GenBank accession numbers GU186708 and AB259712, respectively). To obtain the cDNA of HA_H1_, total RNA was extracted from MDCK cells infected with A/duck/Italy/1447/2005 (H1N1) and reverse transcribed with the RevertAid reverse transcriptase (MBI Fermentas) using the Uni12 oligonucleotide primer [29]. The cDNA of viral RNA segment 4 was amplified with universal primers as described previously [30], ligated into the pJET2.1 plasmid (MBI Fermentas) and sequenced (EMBL/GenBank accession number HF563054). Chimeric H5/H1 and H1/H5 hemagglutinins were constructed with the cDNAs of A/chicken/Yamaguchi/7/2004 (H5N1) and A/duck/Italy/1447/2005 (H1N1) according to a published strategy [31]. The HA_H5_ and HA_H1_ globular head domains comprising amino acids 42–274 (numbering based on the mature HA_H5_ protein) were assembled into the heterologous HA_H1_ and HA_H5_ backbones using fusion PCR technology [32].

For generation of recombinant VSV replicon particles, HA genes were amplified by PCR and inserted into the pVSV* plasmid using single *Mlu*I and *Bst*EII restriction sites upstream and downstream of the fourth transcription unit, thereby replacing the VSV G gene [21]. The resulting plasmids are referred to as pVSV*ΔG(HA_H5-HP_), pVSV*ΔG(HA_H5-LP_), pVSV*ΔG(HA_H1_), pVSV*ΔG(HA_H5/H1_), and pVSV*ΔG(HA_H1/H5_). HA sequences were confirmed by Sanger sequencing.

### Generation of recombinant VSV replicon particles

VSV replicon particles (VRPs) were generated as described previously [33]. Briefly, BHK-G43 cells were infected with recombinant MVA-T7 virus expressing T7 RNA polymerase [34] and subsequently transfected with pVSV*ΔG(HA) along with three plasmids driving T7 RNA polymerase-mediated expression of the VSV proteins N, P, and L. Expression of the VSV G protein was induced by adding mifepristone (Sigma; final concentration 10^−9^ M) to the cell culture medium. At 24 hours post transfection, the cells were detached with trypsin, seeded into T-75 flasks along with an equal number of fresh BHK-G43 cells, and further incubated at 37°C for 24 hours in the presence of mifepristone. The cell culture supernatant was clarified by low-speed centrifugation and passed through a 0.20 µm pore filter. The replicon particles were propagated on mifepristone-induced BHK-G43 cells and stocks were stored at −70°C. All VRPs were titrated on BHK-21 cells in 96-well microtiter plates. Infectious titers were expressed as fluorescence-forming units per milliliter (ffu/ml).

Inactivation of VRPs was performed with a GS Gene Linker UV Chamber (Bio-Rad). A volume of 1 ml of VRP stock was placed into a 35-mm dish and irradiated with UV light (365 nm) corresponding to a total energy of 1.0 Joule. To check for successful inactivation, UV light-treated and non-treated VRPs were added to BHK-21 cells for 8 hours and eGFP expression monitored by fluorescence microscopy.

### Immunofluorescence and flow cytometric analysis

Vero cells grown on 12-mm-diameter cover slips were inoculated for 90 minutes with either VSV*ΔG, VSV*ΔG(HA_H5-HP_) or VSV*ΔG(HA_H5-LP_) using a multiplicity of infection (MOI) of 3 ffu/cell, and further incubated at 37°C. In some experiments, the cells were treated with trypsin and exposed to pH 5.4 prior to immunofluorescence analysis (see below). Cells were fixed at 6–8 hours p.i. with 3% paraformaldehyde for 20 minutes and then washed with PBS containing 0.1 M glycine. The cells were subsequently incubated with swine anti-H5N1 serum (1∶250; kindly provided by Lisa Harwood, IVI Mittelhäusern, Switzerland) and anti-swine IgG antibody conjugated with rhodamine (1∶500; Rockland, Gilbertsville, PA). Finally, the cells were counterstained for 5 minutes at 37°C with 4′,6-diamidino-2-phenylindole (DAPI; Sigma; 0.1 µg/ml in ethanol), washed with distilled water, and embedded in Mowiol 4–88 (Sigma) mounting medium.

For flow cytometric analysis of HA expression, BHK-21 cells were infected with recombinant VRPs using an MOI of 3 ffu/cell. The cells were suspended at 6 hours p.i. in PBS containing 0.5% bovine serum albumin, incubated for 20 minutes at 4°C with chicken anti-H1 or anti-H5 immune serum (1∶100 each), and washed with PBS. Cells were incubated for 20 minutes at 4°C with goat anti-chicken IgY secondary antibody conjugated with Alexa 546 (1∶200, Life Technologies, Carlsbad, CA). The cells were washed and fluorescence was measured with a FACSCalibur cytometer (Becton Dickinson, Franklin Lakes, NJ) and analyzed with the FlowJo software (Treestar Inc., Ashland, OR).

### Western blot analysis

Confluent MDCK cells in T-75 cell culture flasks were inoculated for 90 minutes at 37°C with 10 ml of MEM containing 10^6^ TCID_50_ of A/duck/Hokkaido/Vac-1/2004 (H5N1) and further incubated in serum-free MEM in the presence of 1 µg of acetylated trypsin/ml. At 24 hours p.i., the cell culture supernatant was harvested and clarified by low-speed centrifugation. Influenza virus was pelleted through a 25% (w/w) sucrose cushion by ultracentrifugation at 105,000× g (60 minutes, 4°C). The virus pellet was suspended in 100 µl of milliQ water and the protein content determined with the BCA protein assay kit (Pierce – Thermo Fisher Scientific). Virus proteins were dissolved in sodium dodecyl sulfate (SDS) sample buffer with or without 5% (v/v) β-mercaptoethanol, separated (2 µg protein/lane) by SDS 10% polyacrylamide gel electrophoresis (PAGE), and transferred to nitrocellulose membranes by semi-dry blotting [35]. The nitrocellulose membranes were blocked overnight at 4°C with Odyssey Blocking Reagent (LI-COR Biosciences, Lincoln, NE) diluted 1∶2 with PBS. For immunodetection, the membranes were subsequently incubated with primary (chicken immune sera, 1∶5000) and secondary antibodies (IRDye 800CW donkey anti-chicken IgY, 1∶10,000), diluted in Odyssey Blocking Reagent/PBS (1∶2). The membranes were washed with PBS supplemented with 0.1% Tween-20 and finally with detergent-free PBS. The Western blots were imaged with the Odyssey Infrared Imaging System (LI-COR).

### Animal trials

Specific pathogen-free (SPF) white Leghorn chickens were obtained from the Institute of Virology and Immunology (IVI, Mittelhäusern, Switzerland) breeding flock. Animals (n = 5) were immunized intramuscularly (i.m.) 4 weeks after hatch by injecting cell culture supernatant containing the indicated VRPs (4×10^8^ ffu/ml or less) into both the left and right breast muscle (250 µl each). The animals were kept for 3 weeks employing deep litter management and “*ad libitum*” access to feed and water. At 7 weeks of age, chickens were either immunized a second time or were left untreated. The chickens were challenged at 9 weeks of age with A/whooper swan/Mongolia/3/2005 (H5N1) via the nasal route using 10^6^ TCID_50_ diluted in 100 µl PBS. Following infection, the animals were kept in cages (5 animals per cage) and surveyed daily for clinical signs of disease. A clinical scoring system was used as described previously [21]. At 14 days post infection, all surviving animals were euthanized. For transmission studies, 3 sentinel animals were housed together with 3 infected chickens starting from day 1 post infection (two cages with total 6 primary infected and 6 contact chickens were used for each transmission experiment). All experiments with HPAIV H5N1 were performed in compliance with biosafety level 3.

### Whole virus inactivated vaccine

The LPAIV A/duck/Hokkaido/Vac-1/2004 (H5N1) was passaged in MDCK cells in the presence of acetylated trypsin (1 µg/ml). The virus was pelleted from the cell culture supernatant by ultracentrifugation (105,000× g, 60 minutes, 4°C), suspended in PBS and incubated with paraformaldehyde (0.5% final concentration) overnight at 4°C. The virus preparation was diluted with 25 volumes of PBS containing 0.1 M glycine, pelleted by ultracentrifugation as above and suspended in PBS. The total protein content was estimated by the BCA protein assay kit (Pierce). Equal volumes of inactivated virus suspension and ABM-S adjuvant (Linaris, Bettingen, Germany) were mixed and directly used for intramuscular immunization of chickens. Each animal received 2 injections (250 µl each) corresponding to 5 µg total protein. After 3 weeks, the animals were boosted with the same vaccine and dose.

### Analysis of virus shedding by qRT-PCR

Oropharyngeal and cloacal swabs were daily collected from infected chickens for seven days p.i., suspended in 2 ml of MEM medium and stored at −70°C. Total RNA was extracted from the samples using the NucleoSpin 96 Virus kit (Macherey-Nagel AG, Düren, Germany). For detection of viral RNA, a quantitative real-time RT-PCR based on the amplification of the conserved viral RNA segment 7 was performed in triplicates employing eGFP as internal control [36], [37].

### Serological tests

Hemagglutination (HA), hemagglutination inhibition (HI), and virus neutralization tests were performed according to guidelines of the OIE World Organization of Animal health (http://web.oie.int/eng/normes/en_mmanual.htm). Detection of anti-HA_H5_ antibodies by cELISA was performed using the FlockCheck® kit according to the manufacturer's protocol (IDEXX Laboratories, Liebefeld, Switzerland). Anti-NP antibodies were detected with a commercially available cELISA (ID-Vet, Montpellier, France).

### Syncytia formation assay

Vero cells were grown on glass cover slips (12-mm in diameter) and infected with either VSV*ΔG(HA_H5-HP_), VSV*ΔG(HA_H1_), VSV*ΔG(HA_H1/H5_), or VSV*ΔG(HA_H5/H1_) using an MOI of 1 ffu/cell. At 5 hours p.i. with either VSV*ΔG(HA_H1_) or VSV*ΔG(HA_H5/H1_), cells were treated for 1 hour at 37°C with trypsin (10 µg/ml) in order to activate HA. At 6 hours p.i., all infected cells were incubated for 30 minutes at 37°C with serially diluted chicken immune sera (heat-inactivated for 30 minutes at 56°C) or monoclonal antibody clone C179 (Takara Bio Europe/SAS, Saint-Germain-en-Laye, France) and subsequently exposed for 5 minutes to pH 5.4 in order to trigger the fusion process. Subsequently, cells were incubated in DMEM with 5% FBS for 2 hours at 37°C and fixed overnight at 4°C with 3% paraformaldehyde in PBS. Finally, the nuclei were stained with DAPI (5 minutes, 37°C), washed with distilled water, and embedded in Mowiol 4–88 mounting medium. Infected cells were imaged using an inverted fluorescence microscope (Cell Observer, Zeiss, Jena, Germany). For each experiment a total of 300 to 400 nuclei were counted. The number of nuclei in syncytia per total number of nuclei was calculated and expressed as percent fusion rate (%). The quotient formed by the fusion rate in the presence of HA-specific antibody and the fusion rate in presence of pre-immune serum was calculated and expressed as percent fusion inhibition rate (%).

### Statistical analysis

Mean values and standard deviations were calculated where indicated. Statistical analysis was performed using the unpaired t-test. P<0.05 was considered significant.

## Results

### Generation of RNA replicon particles expressing functionally active H5 hemagglutinin

**Figure 1:**
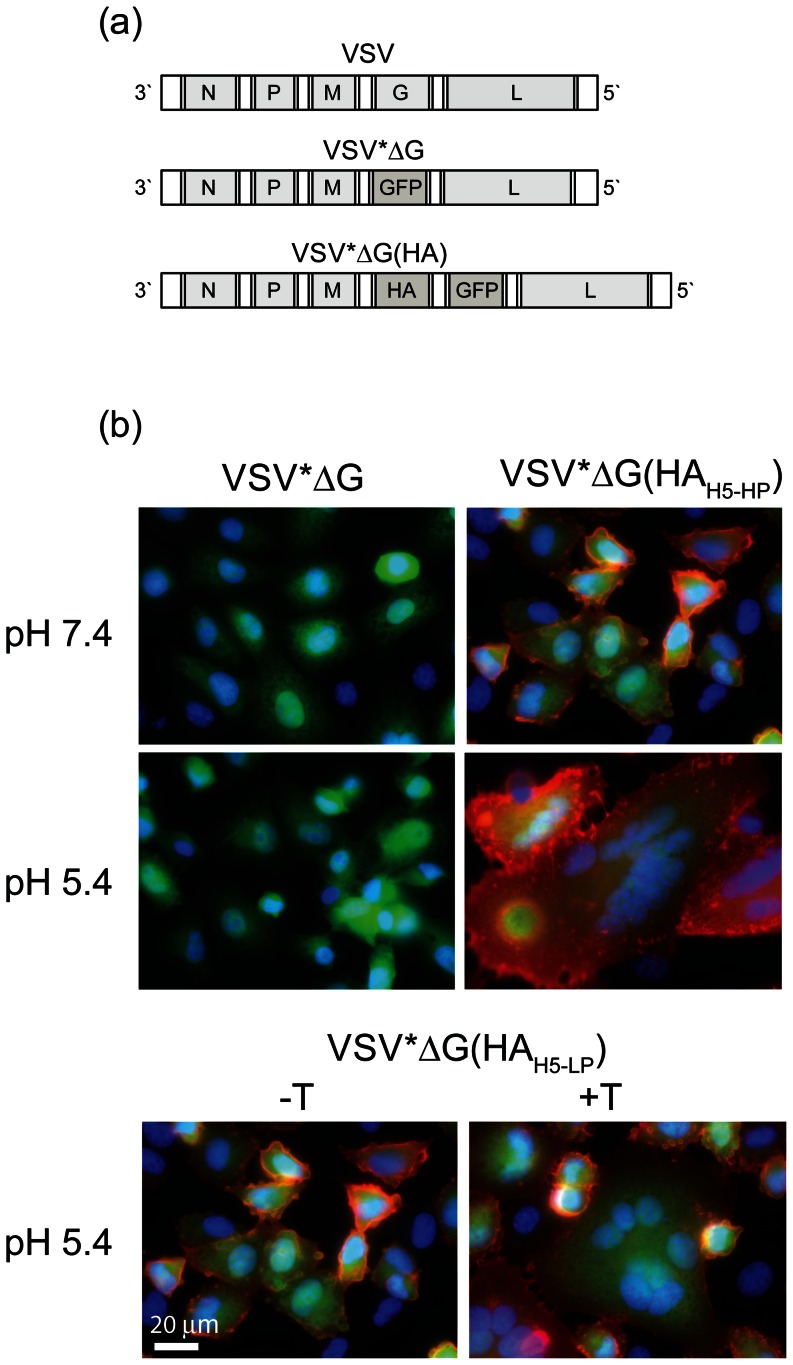
Expression of functional hemagglutinin (HA) with recombinant VRPs. (a) Genome maps of recombinant VSV: the parental VSV genome contains five transcription units encoding for the nucleoprotein (N), phosphoprotein (P), matrix protein (M), glycoprotein (G), and the large RNA polymerase (L). VSV*ΔG lacks the glycoprotein G gene but contains the eGFP gene instead (the asterisk denotes for eGFP). VSV*ΔG(HA) expresses the influenza virus HA from the fourth gene position while eGFP is expressed from an additional transcription unit downstream of HA. (b) Immunofluorescence analysis of Vero cells 8 hours p.i. with either VSV*ΔG, VSV*ΔG(HA_H5-HP_) or VSV*ΔG(HA_H5-LP_). At 5 hours p.i. with VSV*ΔG(HA_H5-LP_), the cells were treated for 60 minutes with trypsin (+T) or were left untreated (−T). Thereafter, the cells were exposed for 5 minutes to either pH 5.4 or pH 7.4, incubated for 60 minutes at 37°C with normal medium, fixed with formalin, and finally processed for immunofluorescence using a swine antiserum to HA_H5_ (red fluorescence). Nuclei were stained with DAPI (blue fluorescence). Expression of eGFP is indicated by green fluorescence. Scale bar represents 20 µm.

Non-transmissible vesicular stomatitis virus (VSV) vectors were generated by replacing the VSV glycoprotein G gene with either the hemagglutinin (HA) gene of A/chicken/Yamaguchi/7/2004 (H5N1), a highly pathogenic avian influenza virus (HPAIV), the HA gene of A/duck/Hokkaido/Vac-1/2004 (H5N1), a low-pathogenic avian influenza virus (LPAIV) [24], or the enhanced green fluorescent protein (eGFP) gene (**Fig. 1a**). To facilitate virus detection and titration, the eGFP reporter protein was expressed from an additional transcription cassette downstream of the HA genes. The resulting viruses, VSV*ΔG(HA_H5-HP_), VSV*ΔG(HA_H5-LP_), and VSV*ΔG were propagated on helper cells providing the VSV G glycoprotein *in trans*. Virus titers of 2–5×10^8^ fluorescence forming units (ffu) per ml of cell culture supernatant were usually attained. The trans-complemented particles were able to infect a broad spectrum of different avian and mammalian cell lines [33]. However, in accordance with our previous observation that HA does not substitute for VSV G protein functions [21], these cells did not release progeny virus (data not shown). We therefore refer to the VSV G protein-complemented, propagation-incompetent viral vectors as to virus replicon particles (VRP).

To study the expression of recombinant HA, Vero cells were infected with VRPs and analyzed by immunofluorescence using swine anti-HA_H5_ immune serum. HA was detected at the cell surface 6 hours post infection (p.i.) with either VSV*ΔG(HA_H5-HP_) or VSV*ΔG(HA_H5-LP_), whereas VSV*ΔG-infected control cells remained negative for HA (**Fig. 1b**). When cells infected with VSV*ΔG(HA_H5-HP_) were briefly exposed to pH 5.4, they started to form large syncytia as a consequence of HA-mediated cell-cell fusion. In case of VSV*ΔG(HA_H5-LP_), fusion did not take place unless the cells were treated with exogenous trypsin (**Fig. 1b** **, lower panel**). These results indicate that the recombinant HAs were expressed at the cell surface in their functionally active conformation.

### VSV*ΔG(HA) protects chickens from lethal infection with heterologous H5N1

To assess whether vaccinated animals would be protected against challenge infection with highly pathogenic H5N1 virus, specific pathogen-free (SPF) chickens at 4 weeks of age were immunized intramuscularly (i.m.) with adjuvant-free VSV*ΔG(HA_H5-HP_) particles using doses of 2×10^6^, 2×10^7^, or 2×10^8^ ffu. Control animals received VSV*ΔG particles (2×10^8^ ffu). A second control group was immunized with UV light-irradiated VSV*ΔG(HA_H5-HP_). At 7 weeks of age, the chickens received a second dose of the vaccine or were left untreated. No adverse effects due to vaccination were observed. Sera were collected from chickens at 9 weeks of age and were tested for virus neutralizing antibodies against A/whooper swan/Mongolia/3/2005/ (H5N1) (**Table 1**). Sera from chickens that had been vaccinated with the control vector VSV*ΔG remained negative for neutralizing antibodies. In contrast, all chickens immunized once with 2×10^8^ ffu of VSV*ΔG(HA_H5-HP_) produced neutralizing antibodies. Antibody titers ranged from 140 to 1350 ND_50_/ml. After booster immunization, serum antibody titers increased significantly (p<0.05) and ranged between 9050 and 51200 ND_50_/ml, indicating that a second vaccine application can effectively strengthen the immune response. In contrast, chickens immunized twice with UV light-treated VSV*ΔG(HA_H5-HP_) did not respond, indicating that replication/transcription of the replicon genome is crucial for triggering the immune response. When chickens were immunized twice with a tenfold lower dose (2×10^7^ ffu) of VSV*ΔG(HA_H5-HP_), only 2 out of 5 chickens showed neutralizing antibody titers above 100 ND_50_/ml. All animals receiving a single immunization with 2×10^7^ ffu or 2 immunizations with 2×10^6^ ffu had antibody titers below 100 ND_50_/ml. These findings suggest that the VRP-based vaccine operates in a dose-dependent manner and has a strong boosting effect if applied a second time.

**Table 1:** Virus neutralizing activity of serum antibodies from vaccinated SPF chickens.

			ND_50_/ml^b^				
			Chicken no.				
VRP^a^	Number of immunizations	Vaccine dose (ffu)	#1	#2	#3	#4	#5
VSV*ΔG	2	2×10^8^	<100	<100	<100	<100	<100
VSV*ΔG(HA_H5-HP_) (UV-irradiated)	2	-	<100	<100	<100	<100	<100
VSV*ΔG(HA_H5-HP_)	1	2×10^8^	1350	400	141	283	200
	2	2×10^8^	12800	51200	9050	9050	9050
VSV*ΔG(HA_H5-HP_)	1	2×10^7^	<100	<100	<100	<100	<100
	2	2×10^7^	283	141	<100	<100	<100
VSV*ΔG(HA_H5-HP_)	2	2×10^6^	<100	<100	<100	<100	<100

a SPF chickens were immunized (i.m.) once or twice with the indicated doses of either VSV*ΔG or VSV*ΔG(HA_H5-HP_).

b Blood was collected from vaccinated chickens at 9 weeks of age.

Serum was prepared and inactivated for 30 minutes at 56°C. Virus neutralisation assays were performed with HPAIV A/whooper swan/Mongolia/3/2005 (H5N1).

**Figure 2:**
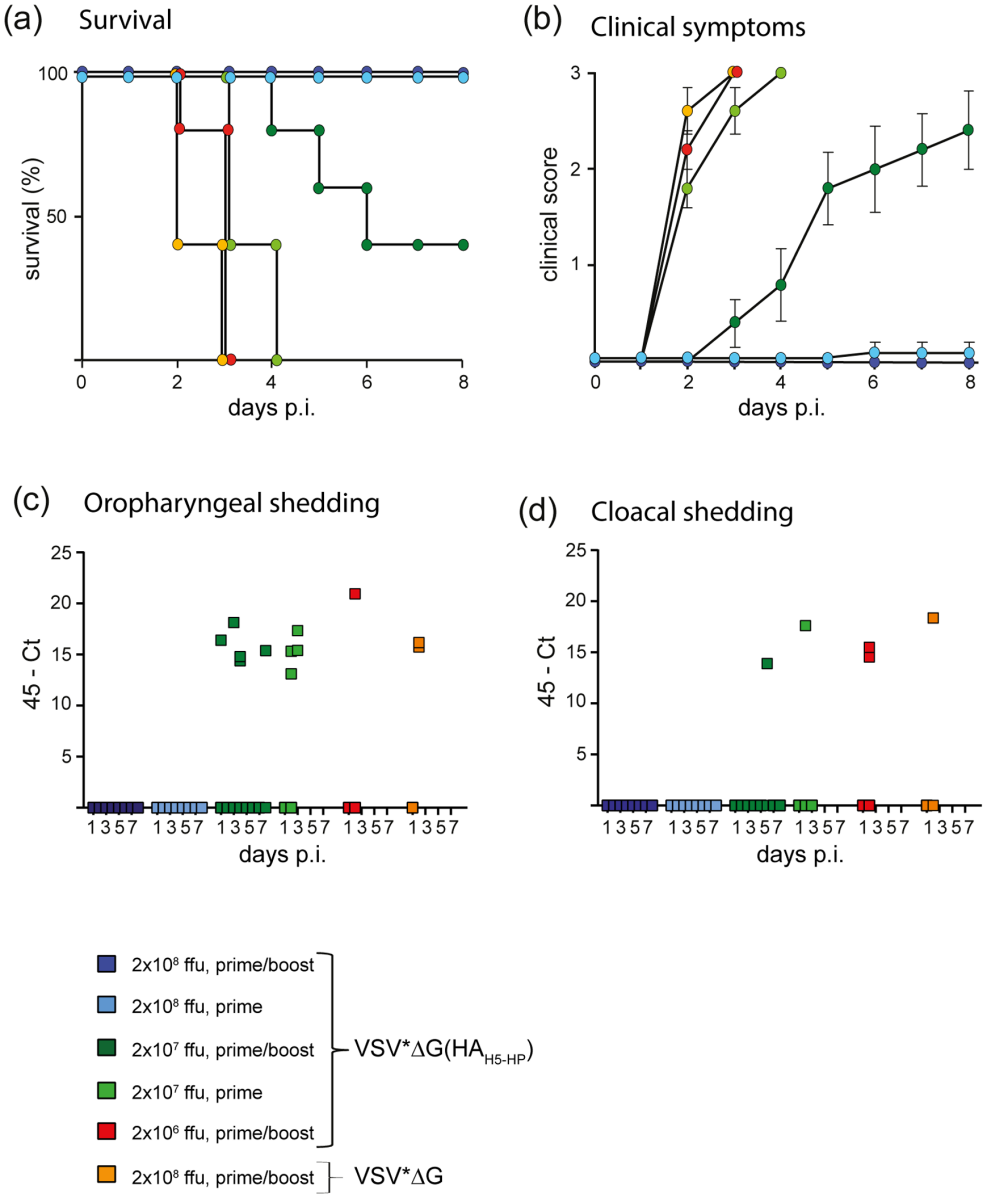
VSV replicon vaccines protect chickens from HPAIV challenge in a dose-dependent manner. Chickens (group size n = 5) were immunized (i.m.) once or twice with VSVΔG(HA_H5-HP_) using the indicated doses and subsequently challenged by intranasal inoculation with 10^6^ TCID_50_ of A/whooper swan/Mongolia/3/2005 (H5N1). The animals were surveyed daily for survival (a) and clinical symptoms (b). The animals were scored as follows: (0) healthy, (1) ill (animals show one of the following symptoms: apathy, ruffled feather, anorexia, diarrhea, cyanosis of the exposed skin, comb and wattles, edemas of the face and/or head, neurological symptoms), (2) severely ill (severe or more than one of the symptoms mentioned above), and (3) dead. A daily clinical index representing the mean value of all chickens per group was calculated. For analysis of virus shedding oropharyngeal (c) and cloacal (d) swabs were taken from each animal at daily intervals for a period of 8 days post infection. RNA was extracted from the swabs and analyzed for the presence of viral RNA segment 7 by quantitative RT-PCR.

Immunized chickens were infected via the intranasal route with 10^6^ TCID_50_ of HPAIV A/whooper swan/Mongolia/3/2005 (H5N1). All animals immunized with VSV*ΔG succumbed to infection (mean time to death 2.3 d) (**Fig. 2a**). Likewise, chickens were not protected if they were vaccinated with lower doses of VSV*ΔG(HA_H5-HP_), i.e. 2 immunizations with 2×10^6^ ffu or a single immunization with 2×10^7^ ffu. The mean times to death were 2.8 d and 3.4 d, respectively. Animals vaccinated twice with 2×10^7^ ffu were partially protected (mean time to death 5 d). Surviving animals in this group showed signs of disease (**Fig. 2b**). In contrast, all animals immunized with 2×10^8^ ffu of VSV*ΔG(HA_HA-HP_), either once or twice, were completely protected and did not show any clinical symptoms (**Fig. 2a, b**). This indicates that immunization with a single high-dose VRP vaccine provides a more robust protection than a low-dose vaccine applied twice.

Analysis of oropharyngeal and cloacal swabs by quantitative RT-PCR showed that secretion of challenge virus was abolished when the chickens received a high dose (2×10^8^ ffu) of VSV*ΔG(HA_HA-HP_) (**Fig. 2c,d**). A single application of the vaccine appeared to be as efficient as a prime/boost application. In contrast, lower vaccine doses did not prevent virus shedding even when applied twice. In order to confirm these results, virus transmission experiments employing naive contact chickens were performed (**Table 2**). At first, chickens were vaccinated twice with 2×10^8^ ffu of either VSV*ΔG or VSV*ΔG(HA_HA-LP_) and challenged with A/whooper swan/Mongolia/3/2005 (H5N1). At day 1 post infection, naive contact chickens were co-housed with the infected animals. Sentinel chickens died 2 days later if they were kept together with VSV*ΔG-immunized chickens, indicating that challenge virus was efficiently transmitted from the control animals. In contrast, all sentinels housed together with VSV*ΔG(HA_H5-LP_)-vaccinated chickens remained healthy and did not seroconvert to influenza virus NP antigen. Infected animals were easily discriminated from vaccinated ones by employing two commercially available ELISA tests for the detection of NP and HA antibodies, respectively (**Table 2**). Although vaccinated animals were clinically protected from challenge infection and did not secrete virus, five of six animals seroconverted to NP antigen, indicating that limited virus replication occurred.

**Table 2:** Survival rate and seroconversion of vaccinated and sentinel SPF chickens following challenge infection with A/whooper swan/Mongolia/3/2005 (H5N1).

		Survival rate and seroconversion of chickens^a^			
		Transmission experiment 1		Transmission experiment 2	
		VSV*ΔG	sentinel	VSV*ΔG (HA_H5-LP_)	sentinel
Survival		0	0	6	6
Sero-conversion (NP)	pre- challenge	0	0	0	0
	post- challenge	NA	NA	5	0
Sero-conversion (HA_H5_)	pre- challenge	0	0	6	0
	post- challenge	NA	NA	6	0

a SPF chickens (n = 6) were immunized twice with either VSV*ΔG or VSV*ΔG(HA_H5-LP_) and subsequently challenged with A/whooper swan/Mongolia/3/2005 (H5N1).

At 1 day post challenge, sentinel birds (n = 6) were housed along with the infected chickens. The animals were serologically tested directly before infection and 12 days post infection for the presence of NP and HA_H5_ antibodies, respectively. The number of surviving and seropositive animals are shown. NA, not applicable.

### VSV*ΔG(HA) induces antibodies with broadly neutralizing activity

Neutralizing antibodies often interfere with the receptor-binding activity of HA by binding to epitopes in the globular head domain [38]. Minor changes in these epitopes may affect binding of antibodies resulting in reduced or even total loss of neutralizing activity. To assess the range of neutralizing activity of chicken immune sera, we performed virus neutralization tests with a panel of H5 viruses belonging to different phylogenetic clades. Chickens vaccinated with VSV*ΔG(HA_H5-HP_) developed antibodies directed against the HA_H5_ antigen of A/chicken/Yamaguchi/7/2004 (clade 2.5), which efficiently neutralized all H5 isolates tested (**Table 3**). Chickens immunized with VSV*ΔG(HA_H5-LP_) produced antibodies directed to the HA_H5_ antigen of A/duck/Hokkaido/Vac-1/2004 (classical clade). These antibodies were also capable of neutralizing the whole spectrum of H5 isolates. However, viruses of clades 1, 2.2, and 2.5 were neutralized with lower efficacy than viruses belonging to the classical clade (p<0.05). Compared to the VSV*ΔG(HA_H5-LP_) vaccine, the inactivated A/duck/Hokkaido/Vac-1/2004 vaccine induced even lower levels of neutralizing antibodies against viruses of clades 1, 2.2, and 2.5. Interestingly, immune sera from chickens vaccinated with live A/duck/Hokkaido/Vac-1/2004 neutralized all viruses of the classical clade but failed to neutralize viruses belonging to clades 1, 2.2, or 2.5. This indicates that A/duck/Hokkaido/Vac-1/2004 HA_H5_ is antigenically distinct and less related to the HA_H5_ antigen of clades 1, 2.2, or 2.5.

**Table 3:** Virus neutralizing activity of immune sera from SPF chickens following vaccination with either VRPs, inactivated virus, or live attenuated virus.

		Virus neutralization titers (ND_50_/ml) following immunization with^a^			
Virus	Clade	VSV*ΔG (HA_H5-HP_)	VSV*ΔG (HA_H5-LP_)	Inactivated Vac-1/04 (H5N1)	Live Vac-1/04 (H5N1)
A/Vietnam/1194/2004 (H5N1)	1	2'260	2'260	560	<100
A/chicken/Yamaguchi/7/2004 (H5N1)	2.5	12'800	1'350	670	<100
A/whooper swan/Mongolia/3/2005 (H5N1)	2.2	6'400	1'350	470	<100
A/Cygnus olor/Italy/742/2006 (H5N1)	2.2	10'800	4'550	280	<100
A/duck/Hokkaido/Vac-1/2004 (H5N1)	-	21'300	72'800	72'400	21'500
A/duck/Potsdam/1402/1986 (H5N2)	-	9'050	30'800	6'400	800
A/duck/Potsdam/2216/84 (H5N6)	-	5'400	21'300	4'530	1'130
A/duck/Potsdam/619/85 (H5N2)	-	10'750	36'200	21'520	2'260
A/ostrich/Germany/R5-10/03 (H5N2)	-	16'400	15'750	21'280	1'640
A/mallard/Föhr/Wv1310-13/03 (H5N2)	-	10'800	18'100	9'050	960
A/teal/Föhr/Wv1378-79/03 (H5N2)	-	3'800	6'400	21'528	1'340

a SPF chickens were immunized with four different vaccines.

VSV*ΔG(HA_H5-HP_) and VSV*ΔG(HA_H5-LP_) were applied intramuscularly without adjuvant using 2×10^8^ ffu for both the primary and booster immunization. Inactivated A/duck/Hokkaido/Vac-1/04 (H5N1) was formulated with adjuvant and applied two times via the intramuscular route. Live A/duck/Hokkaido/Vac-1/04 (H5N1) was applied one time via the intratracheal route using 10^7^ TCID_50_. Serum was prepared from 4 to 5 animals per group and pooled. Virus neutralization tests were performed with the viruses listed in the left column. Neutralization titers obtained with homologous virus are underlined.

### Chicken immune sera bind to both HA subunits

**Figure 3:**
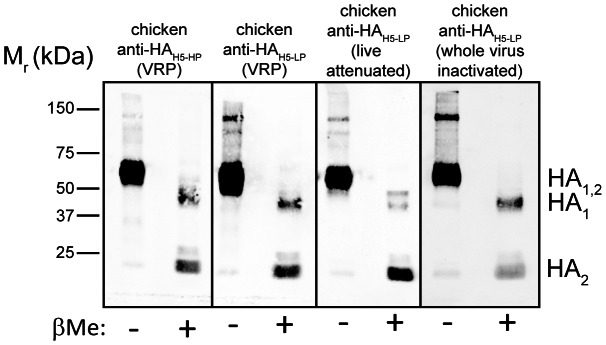
Western-blot analysis of purified LPAIV particles with different chicken immune sera. A/duck/Hokkaido/Vac-01/2004 (H5N1) was grown on MDCK cells in the presence of trypsin and pelleted from the cell culture supernatant by ultracentrifugation. The virus particles were separated by SDS-PAGE under non-reducing (− βME) or reducing (+ βME) conditions and blotted onto a nitrocellulose membrane. HA antigen was detected with the chicken immune sera indicated on top of the blot. The positions of marker proteins of known molecular masses are indicated on the left, the positions of the HA subunits on the right hand side.

Neutralizing antibodies may be directed either to the globular head domain, which is part of the HA_1_ subunit, or to the conserved stalk domain [39]. To see which part of the HA molecule is recognized by the chicken immune sera, the A/duck/Hokkaido/Vac-1/2004 (H5N1) vaccine strain was grown on MDCK cells in the presence of trypsin, pelleted from the cell culture supernatant by ultracentrifugation, and separated by SDS polyacrylamide gel electrophoresis under non-reducing as well as reducing conditions. Western blot analysis with chicken immune sera from all four vaccine groups showed that the subunits HA_1_ and HA_2_ were recognized equally well (**Fig. 3**). Quantitative fluorescence signals showed that the chicken immune sera bound with higher affinity (approx. 4-fold) to the non-reduced HA than to the reduced one. The intact disulfide bonds most likely prevented entire denaturation of HA, facilitating the binding of conformation-dependent antibodies.

### Chicken immune sera inhibit fusion activity of chimeric HA

**Figure 4:**
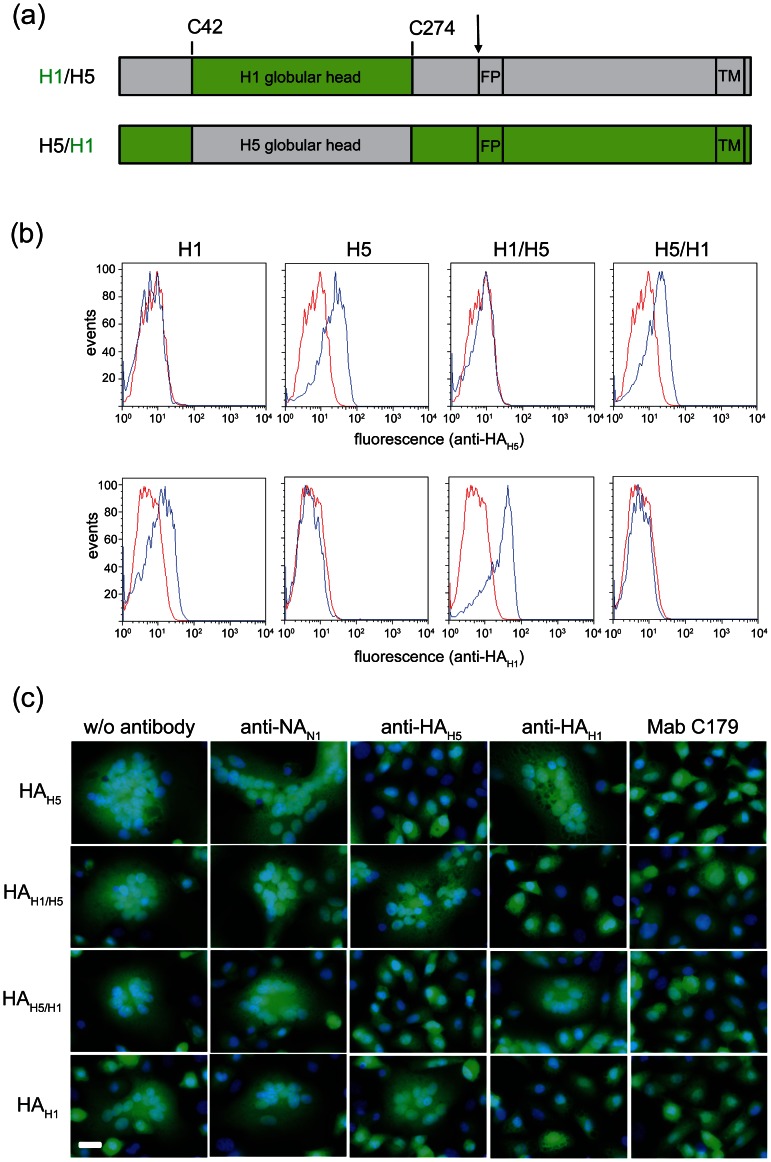
Inhibition of HA-mediated syncytia formation by chicken immune sera. (a) Diagrams of chimeric hemagglutinins. The chimeric H1/H5 hemagglutinin was constructed by swapping the globular head domain located between C42 and C274 of A/chicken/Yamaguchi/8/2004 (H5N1) HA_H5_ (grey) with that of the corresponding region from A/duck/Italy/1447/2005 (H1N1) HA_H1_ (green). The chimeric H5/H1 hemagglutinin was constructed accordingly by replacing the HA_H1_ globular head domain with the corresponding HA_H5_ domain. The proteolytic cleavage site (arrow), fusion peptide (FP) and transmembrane (TM) domain are indicated. (b) Flow cytometric analysis of BHK-21 cells expressing parental and chimeric HAs. Cells were infected with either VSV*ΔG (blue graphs) or VRPs expressing the indicated HAs (red graphs). At 6 hours p.i., cells were stained with chicken immune sera specific for either HA_H5_ or HA_H1_ and goat anti-chicken IgY Alexa-546 conjugates. (c) Inhibition of syncytia formation. Vero cells were infected with VRPs expressing the indicated HAs using an MOI of 5 ffu/cell. At 5 hours p.i., cells were treated for 60 minutes with acetylated trypsin to proteolytically activate HA_H1_ and HA_H5/H1_. At 6 hours p.i., the cells were incubated for 30 minutes at 37°C with the indicated chicken immune sera (diluted 1∶20 in MEM), exposed for 5 minutes at 37°C to pH 5.4, further incubated in medium for 2 hours, and fixed with paraformaldehyde. The nuclei were stained with DAPI (blue fluorescence). Expression of eGFP is indicated by green fluorescence. Scale bar represents 20 µm.

In order to map the neutralizing activity of chicken immune sera to either the globular head or the stalk region of HA, chimeric HA_H1/H5_ was constructed by replacing the globular head region in the HA of A/chicken/Yamaguchi/7/2004 (H5N1) with the corresponding region from A/duck/Italy/1447/2005 (H1N1) (**Fig. 4a**). The complementary HA_H5/H1_ protein was generated by replacing the HA globular head region from A/duck/Italy/1447/2005 (H1N1) with the corresponding domain from A/chicken/Yamaguchi/7/2004 (H5N1). The chimeric HA genes as well as the authentic HA_H1_ were cloned into the VSV*ΔG vector and VRPs produced on helper cells. Expression of the recombinant HAs was studied in BHK-21 cells 6 hours post infection. Flow cytometric analysis revealed that both HA_H1_ and HA_H1/H5_ were recognized by anti-HA_H1_ but not by anti-H5 serum. Vice versa, anti-HA_H5_ serum bound to HA_H5_ and chimeric HA_H5/H1_ glycoprotein but neither to HA_H1/H5_ nor to HA_H1_ (**Fig. 4b**).

Chicken immune sera were tested for their ability to inhibit low pH-triggered syncytia formation [40]. At first, Vero cells were infected with VRPs expressing either HA_H5-HP_, HA_H1_, HA_H5/H1_, or HA_H1/H5_. In the case of HA_H1_ and HA_H5/H1_, the cells were treated with trypsin 5 hours p.i. in order to proteolytically activate HA at the cell surface. Subsequently, the cells were briefly exposed to pH 5.4 to trigger the fusion process. All four HA glycoproteins were able to induce syncytia, indicating that the chimeric glycoproteins HA_H1/H5_ and HA_H5/H1_ were functionally active (**Fig. 4c**). Fusion activity was not inhibited if the cells were incubated with chicken anti-NA_N1_ immune serum, whereas the cross-subtypic monoclonal antibody C179 [40]–[42] inhibited all four HAs, the authentic as well as the chimeric ones (**Table 4**). Anti-HA_H5_ immune sera from VSV*ΔG(HA_H5-HP_)-vaccinated chickens specifically inhibited syncytia formation mediated by HA_H5-HP_ and HA_H5/H1_, but did not affect fusion mediated by HA_H1/H5_ and HA_H1_ (**Fig. 4c** **,** **Table 4**). Correspondingly, anti-HA_H1_ serum inhibited fusion triggered by HA_H1/H5_ and HA_H1_, whereas this serum did not inhibit the fusion activity of HA_H5-HP_ and HA_H5/H1_. These results suggest that the neutralizing activity of the chicken immune sera tested is predominantly directed against the HA globular head domain. The antigenic sites in this domain have previously been identified [43], [44].We compared the amino acid residues at these sites for 6 different H5 viruses used in this study (**Table 5**). It turned out that low-pathogenic H5 viruses belonging to the classical clade are antigenically more distinct from A/Yamaguchi/7/2004 (clade 2.5) than the viruses of clades 1 and 2.2.

**Table 4:** Fusion inhibitory activity of chicken immune sera.

	Fusion inhibitory dose 50%^a^			
VRP	chicken anti-NA_N1_ (VRP)	chicken anti-HA_H5_ (VRP)	chicken anti-HA_H1_ (VRP)	mouse Mab C179
VSV*ΔG(HA_H5-HP_)	<10	213 (±92)	<10	113 (±41)
VSV*ΔG(HA_H1/H5_)	<10	10 (±10)	60 (±23)	113 (±41)
VSV*ΔG(HA_H5/H1_)	<10	133 (±46)	10 (±10)	226 (±83)
VSV*ΔG(HA_H1_)	<10	<10	120 (±46)	293 (±122)

a Vero cells were infected with the indicated recombinant VRPs using an MOI of 5 ffu/cell.

Five hours p.i., HA_H5/H1_ and HA_H1_ were proteolytically activated with trypsin. Six hours p.i., the cells were incubated with serial dilutions of the indicated chicken sera or Mab C179, shortly exposed to pH 5.4 and incubated for 2 hours in normal medium prior to fixation. The serum dilution causing 50% inhibition of syncytia formation was calculated. Mean values and standard deviations of three independent experiments are shown.

**Table 5:** Genetic polymorphism of HA_H5_ genes in the globular head region.

	Amino acid residue in HA of^b^					
Amino acid position^a^ (antigenic site)	A/chicken/Yamaguchi/7/2004 (H5N1) clade 2.5	A/whooper swan/Mongolia/3/2005 (H5N1) clade 2.2	A/Vietnam/1194/2004 (H5N1) clade 1	A/duck/Hokkaido/Vac-01/2004 (H5N1) classical clade	A/duck/Potsdam/1402-6/1986 (H5N2) classical clade	A/teal/Foehr/Wv1378-79/2003 (H5N2) classical clade
45 (C)	D	D	D	S	S	S
71 (E)	I	L	I	L	L	L
83 (E)	A	I	A	D	D	D
86 (E)	S	A	V	I	I	V
94	N	N	D	D	D	D
124 (B)	D	D	S	D	D	N
129	S	S	L	S	S	S
138 (A)	Q	Q	Q	N	N	N
140 (A)	R	R	K	R	R	R
155 (B)	S	N	S	N	N	S
156	A	A	T	A	A	A
174	V	V	V	V	I	V
181	P	P	P	P	P	P
183	D	D	D	D	D	D
189 (B)	R	R	K	K	K	K
212 (D)	K	K	R	E	E	E
227	E	E	E	E	E	E
263 (E)	A	T	T	T	T	T
269	L	L	L	L	L	L
277	K	K	K	K	K	K
282 (C)	M	I	M	M	M	M

a Numbering according to the mature H5 hemagglutinin.

b EMBL/GenBank accession numbers: A/chicken/Yamaguchi/7/2004 (GU186708), A/whooper swan/3/2005 (BAE48316), A/Vietnam/1194/2004 (ABP51976), A/duck/Hokkaido/Vac-01/2004 (AB259712), A/duck/Potsdam/1402-6/1986 (AAD13574), A/teal/Foehr/Wv1378-79/2003 (HF563058).

## Discussion

The H5N1 epizootics, which started in Guangdong in 1996, has killed or resulted in culling of more than 250 million domestic and wild-living birds in 63 countries [13]. As part of their control strategy, several countries implemented vaccination programs mainly employing inactivated influenza virus vaccines. Although these vaccination programs reduced the burden of H5N1, vaccine failure due to antigenic drift remained a problem [45], [46]. Moreover, secretion of antigen-drifted field virus from vaccinated animals represents a considerable risk for unnoticed virus spread, especially as inactivated vaccines normally do not comply with the DIVA principle. Thus, there is a need for safe and broadly protective DIVA vaccines which can be rapidly adapted if new strains emerge.

Recently, we have demonstrated that propagation-incompetent replicon particles expressing the HA_H7_ antigen of A/chicken/Rostock/8/1934 (H7N1) provided full protection against infection with A/chicken/Italy/445/1999 (H7N1), even though the latter virus was isolated 65 years after the former [21]. In this study, we showed that propagation-incompetent VRPs expressing HA_H5-HP_ (clade 2.5) can confer complete protection from lethal infection with heterologous H5N1 (clade 2.2). In addition, the serum antibodies induced by this vaccine also neutralized A/Vietnam/1194/2004 (clade 1) as well as several low-pathogenic H5 viruses representing the classical clade. Vice versa, VRPs encoding the HA_H5-LP_ antigen of A/duck/Hokkaido/Vac-01/2004 (classical clade) induced serum antibodies capable of neutralizing viruses of clades 1, 2.2, and 2.5, although the vaccine antigen differed from the HA of the latter viruses at several antigenic sites (see Table 5). In contrast, immune sera from chickens immunized with live A/duck/Hokkaido/Vac-01/2004 failed to neutralize viruses of clades 1, 2.2, or 2.5, although it neutralized related viruses of the same clade. This indicates that the VRP vaccine has a higher potential than the live-attenuated vaccine for inducing broadly neutralizing antibodies. Likewise, propagation-incompetent VSV replicon particles expressing HA_H5_ were found to induce broadly neutralizing antibodies in mice, which provided long-term protection against challenge infection with different phylogenetic clades of H5N1 [47]. Future studies must show whether the VRP vaccines will also protect chickens against H5N1 of clades 1.1, 2.3.2.1, 2.3.4, and 7 that are presently circulating in Asia [48].

Vaccines will contribute to the control of H5N1 epizootics only if they are able to significantly reduce virus transmission [49]. This goal may be achieved using inactivated influenza virus vaccines [16], [17], [50]. However, there are also examples showing that inactivated vaccines sometimes fail to prevent virus shedding, especially if birds were immunized only once, or if the vaccine strain did not adequately match the HA antigen of the challenge virus [14], [51]. Interestingly, vaccination of chickens with recombinant VRPs expressing HA_H5-HP_ (clade 2.5) abolished cloacal and oropharyngeal shedding of A/whooper swan/Mongolia/3/2005 (clade 2.2). In addition, vaccination with VRPs expressing HA_H5-LP_ (classical clade) prevented transmission of A/whooper swan/Mongolia/3/2005 to sentinel birds housed in the same cage. In these experiments, animals were immunized twice which resulted in high titers of neutralizing serum antibodies. However, a single vaccine dose was already sufficient to protect chickens against lethal infection with A/whooper swan/Mongolia/3/2005 and to reduce virus shedding, despite significantly lower levels of neutralizing serum antibodies induced. Further studies must show whether transmission to sentinels can be prevented if animals are vaccinated once.

The prophylactic vaccination of poultry against H5 and H7 viruses is not allowed in many countries. One reason for this policy is the difficulty to serologically discriminate between infected and conventionally vaccinated animals (DIVA). The VRP vaccine fully complies with the DIVA principle because it only encodes for the influenza virus HA antigen [21]. This enabled us to distinguish between infected and vaccinated animals by taking advantage of a commercially available cELISA for detection of NP antibodies.

Vaccination with VRPs induced a protective immune response in chickens although adjuvants were not employed. The efficacy of the VRP vaccine may be attributed to three factors (i) efficient delivery of the recombinant RNA to cells, (ii) high level antigen expression, and (iii) cytotoxicity of the vector. The replicon particles are coated with the VSV G protein which mediates binding of the particles to a common receptor, subsequent receptor-mediated endocytosis, and low pH-triggered membrane fusion. In the cytosol, the viral RNA polymerase catalyzes several rounds of replication/transcription of the negative-strand RNA genome resulting in enormous amplification of the genetic information and high-level antigen expression. Replication/transcription of the viral RNA genome is required to trigger the immune system as recombinant VRPs did not induce an antibody response to HA antigen when irradiated with UV light. By virtue of the host shut-off activity of the VSV matrix protein, infected cells are destined for apoptosis [52], [53], which is generally regarded as non-immunogenic [54]. However, if the VRP vaccine is used at high doses apoptotic cells may not be cleared quickly enough, allowing secondary necrosis to take place. Necrotic cells are highly immunogenic, stimulating professional antigen-presenting cells due to the release of proinflammatory cytokines and chemokines [55]. These cells phagocytose necrotic cell debris, proteolytically process the proteins, and finally present peptide epitopes to T lymphocytes. This process may explain why the VRP vaccine - in contrast to many inactivated vaccines - did not rely on adjuvant to be immunogenic. Any risk of adverse effects due to the use of adjuvants is therefore eliminated.

Previous work showed that recombinant HA does not functionally substitute the deleted VSV G protein [21]. Therefore, propagation of the vector in vaccinated animals is not possible and this certainly contributes to the safety of the vaccine. Propagation-incompetent VRP vaccines not only are avirulent, they are also unable to revert to virulence. Another positive consequence of the VSV G deletion is that the immunized animals do not produce antibodies to the VSV G protein which could neutralize the VRPs [47]. This allowed us to use the vaccine in homologous prime-boost protocols. It should also be noted that VSV is not a natural avian pathogen, thus eliminating the problem of preexisting immunity to the vector.

Conventional inactivated influenza virus vaccines for use in poultry are produced in embryonated chicken eggs. In case of a new HPAIV epidemic millions of eggs have to be supplied at short notice, which may be a problem given that eggs represent an important protein source in many countries. In contrast, VRP vaccines are propagated to high titers on helper cells which offers a convenient way to produce vaccines in bulk. In addition, the replicon vector represents a very flexible vaccine platform which allows to instantly switch the antigen if this should be necessary.

The intramuscular route of vaccination with VRPs was used throughout this study. This vaccination turned out to be very effective, however it is not useful for mass vaccinations in poultry industry. Therefore, alternative routes of application are currently being evaluated.
